# Human matriptase/ST 14 proteolytically cleaves H7N9 hemagglutinin and facilitates the activation of influenza A/Shanghai/2/2013 virus in cell culture

**DOI:** 10.1111/irv.12707

**Published:** 2019-12-09

**Authors:** Gary R. Whittaker, Marco R. Straus

**Affiliations:** ^1^ Department of Microbiology and Immunology College of Veterinary Medicine Cornell University Ithaca NY USA

**Keywords:** H7N9, hemagglutinin, influenza, LPAI, matriptase, matriptase/ST 14

## Abstract

**Background:**

Influenza is a zoonotic disease that infects millions of people each year resulting in hundreds of thousands of deaths, and in turn devastating pandemics. Influenza is caused by influenza viruses, including influenza A virus (IAV). There are many subtypes of IAV but only a few seem to be able to adapt to humans and to cause disease. In 2013, an H7N9 IAV subtype emerged in China that does not cause clinical symptoms in its chicken host but leads to severe infections when transmitted into humans. Since 2013, there have been six epidemic waves of H7N9 with 1567 laboratory‐confirmed human infections and 615 deaths. Pathogenicity of IAV is complex, but a crucial feature contributing to virulence is the activation of the hemagglutinin (HA) fusion protein by host proteases that triggers membrane fusion and leads to subsequent virus propagation.

**Methods:**

293T, VERO, and MDCK cells were used to conduct Western blot analysis, immunofluorescence assays, and pseudoparticle and live virus infections, and to evaluate H7N9 HA cleavage‐activation.

**Results/Conclusions:**

We show that human matriptase/ST 14 is able to cleave H7N9 HA. Cleavage of H7N9 HA expressed in cell culture results in fusogenic HA and syncytia formation. In infection studies with viral pseudoparticles carrying matriptase/ST 14‐activated H7N9 HA, we observed a high infectivity of cells. Finally, human matriptase/ST 14 also activated H7N9 live virus which resulted in high infectivity. Our data demonstrate that human matriptase/ST 14 is a likely candidate protease to promote H7N9 infections in humans.

## 1 INTRODUCTION

Influenza A viruses (IAVs) circulate in waterfowl as their natural reservoir and are categorized based on the antigenic properties of their hemagglutinin (HA) and neuraminidase (NA) proteins. There are 16 different HAs and nine different NA present in wild birds.^1^ Frequently, wild birds infect poultry resulting in the culling of millions of birds and significant economic losses. However, wild birds rarely show disease symptoms while IAV in poultry usually exhibits respiratory tropism and is able to mutate into a more highly pathogenic form.^2^ Influenza is also a zoonotic disease, and the virus is occasionally transmitted from infected poultry to humans. Certain HA/NA combinations, however, seem to be more favorable for human infections. H1N1, H2N2, and H3N2 have caused several devastating outbreaks in humans in the past such as the 1918 “Spanish flu,” the 1957 “Asian pandemic,” the 1968 “Hong Kong pandemic,” and the 2009 “Swine flu,” respectively.^3^

Currently, the recently emerged H7N9 strain is of concern for global public health. Low‐pathogenic avian influenza (LPAI) H7N9 was first discovered in humans in 2013 in China when three patients were hospitalized with severe and fatal influenza.^4^ Since then, there have been six epidemic H7N9 waves that resulted in 1567 laboratory‐confirmed human infections and 615 deaths and a seventh wave is currently ongoing.^5^ Intriguingly, the virus isolated from the 2013 outbreak was suggested to be derived from chickens that did not show any (or only very mild) symptoms of disease. Further investigations revealed that the viral genes were exclusively from avian origin.^4^ To this date, there are no reports of H7N9 human to human transmissions even though it cannot be excluded in very few cases. However, most cases of human H7N9 infections were attributed to exposure of humans to infected wild birds or poultry and live animal markets.^5^

A crucial step in the life cycle of H7N9 (and all other influenza subtypes) is the cleavage of its HA fusion protein by host proteases. HA is synthesized as a precursor and requires proteolytic processing to exert its fusogenic activity. Cleavage occurs at the C‐terminus of the fusion peptide and results in its exposure allowing HA to induce fusion of the viral and cellular membrane and subsequent release of the RNA into the host cell.^6^, ^7^ LPAI viruses have a monobasic cleavage site that consists of a single arginine, which is preceded by three amino acids that are influenza subtype‐specific. While LPAI viruses in wild birds are usually cleaved by trypsin in their gastro‐intestinal tract, there are trypsin‐like serine proteases in humans and poultry that reside in the respiratory tract and which are able to process these viruses. Therefore, the disease is usually confined to this tissue.^8^

Only very little is known about the activation of H7N9 viruses. Previously, it was reported that the type II transmembrane serine protease TMPRSS2 plays a role in the cleavage of H7N9 HA and activation of the virus in mice.^9^, ^10^ Both studies showed that TMPRSS2 knockout (KO) mice were highly tolerant to H7N9 infections. When KO mice were complemented with human or mouse TMPRSS2, their susceptibility to the virus was restored.^9^ We recently reported that human matriptase/ST 14 was able to very efficiently cleave a H7N9 cleavage motif peptide mimic.^11^ Matriptase/ST 14 is a membrane‐bound protease localized in epithelial cells of various tissues in humans that seems to play a significant role in the activation of various influenza A subtypes such H1N1 and H9N2.^12^, ^13^ However, results obtained using cleavage site peptide mimics do not always reflect the in vivo situation and need to be validated.^11^, ^14^

Here, we show that human matriptase/ST 14 cleaves H7N9 HA resulting in a fusogenic active protein. We further demonstrate that matriptase/ST 14 promotes the infectivity of pseudoparticles carrying H7N9. Ultimately, we provide data showing the activation of A/Shanghai/2/2013 virus by human matriptase/ST 14 leading to a high infectivity in cell culture. Together, our data suggest that matriptase/ST 14 is a novel protease in the human respiratory tract that promotes H7N9 infection.

## 2 MATERIALS AND METHODS

### 2.1 Cells, viruses, plasmids and protease

293T, VERO, and MDCK cells (American Type Culture Collection) used for influenza experiments were maintained in Dulbecco's modified eagle medium (DMEM) supplemented with 25 mM HEPES (Cellgro) and 10% HyClone FetalClone II (GE). The human isolate of influenza A/Shanghai/2/2013 H7N9 virus was a gift from Stacey Schultz‐Cherry (St. Jude Children's Hospital, Memphis, TN, USA). The virus was propagated in cell culture. In brief, 25 cm^2^ flasks with confluent MDCK cells were inoculated with different dilutions of A/Shanghai/2/2013 H7N9. Cells were monitored daily for cytopathogenic effects. Supernatants were harvested when about 60‐70% of cells/flask were dead. Supernatants were combined, and the viral titer was quantified as described below.

The pDZ plasmids encoding for A/Shanghai/2/2013 H7N9 HA and NA were kindly provided by Dr Adolfo Garcia‐Sastre, Icahn School of Medicine at Mount Sinai, New York City, NY, USA. The pCMV‐MLVgag‐pol murine leukemia virus (MLV) packaging construct, the pTG‐Luc transfer vector encoding luciferase reporter, and pCAGGS/VSV‐G plasmid were described before.^15^, ^16^ Recombinant human matriptase/ST 14 was purchased from R&D systems. The specific activity of the enzyme is described as 10 000 pmol/min/µg for the fluorogenic peptide substrate Boc‐QAR‐AMC.

### 2.2 Matriptase/ST 14 cleavage experiment and western blot analysis

293T cells were grown in 6‐well plates and transfected with pDZ/H7N9 HA and pDZ/H7N9 NA using Turbofect according to the manufacturer's recommendations (Invitrogen). Eighteen hours post–transfection, cells were washed with PBS, and 200 nM matriptase/ST 14 in matriptase buffer (20 mM Tris, 10 mM CaCl_2_, 150 mM NaCl, pH 7.4) was added to one well and incubated on a rocker at 37°C and 5% CO_2_ for 90 minutes. For the control, 0.8 mM Trypsin in PBS was added for 10 minutes. Cells were then processed for Western blot analysis as previously described.^12^ The antibody to detect A/Shanghai/2/2013 H7N9 HA (NR48765) was obtained from the Biodefense and Emerging Infections Research Resources Repository. The secondary antibody had an Alexa fluor 488 tag and was purchased from Invitrogen. Western blot membranes were scanned using a ChemiDoc imaging system (Bio‐Rad) to facilitate exposure in a linear range. Bands were quantified using ImageJ software, and cleavage efficiencies for HA_1_ were calculated by the following equation: (HA_1_/[HA_0_ + HA_1_])*100. Graph was generated using Graphpad Prism 7 software.

### 2.3 Cell‐cell fusion assay

VERO cells were grown in 24‐well plates that contained a glass cover slide and were transfected with pDZ/H7N9 HA and pDZ/H7N9 NA using Lipofectamine 2000 according to the manufacturer's recommendation (Invitrogen). Eighteen hours post‐transfection, cells were washed, and 200 nM matriptase/ST 14 in matriptase buffer was added and incubated on a rocker for 3 hours. Control cells were incubated with 0.8 nM Trypsin in PBS for 10 minutes. Cell‐cell fusion and immunofluorescence assay was performed as previously described.^12^

### 2.4 Pseudoparticle production and infection

For pseudoparticle production, 293T cells were grown in 6‐well plates and transfected with pDZ/H7N9 HA, pDZ/H7N9 NA, pCMV‐MLVgag‐pol, and pTG‐Luc using Turbofect according to the manufacturer's recommendations. Controls were transfected with pCAGGS/VSV‐G, pCMV‐MLVgag‐pol, and pTG‐Luc (positive control) or only pCMV‐MLVgag‐pol and pTG‐Luc (negative control). Supernatants were harvested 72 hours post‐transfection, and pseudoparticles were stored at −80°C. Infections, cell lysis, and data analysis were performed as previously described.^17^ Experiment was repeated three independent times. Results were plotted, and statistical analysis was conducted using Graphpad Prism7 software.

### 2.5 Virus infections and quantification

All experiments with A/Shanghai/2/2013 virus were conducted under biosafety level 3 (BSL3) conditions. MDCK cells were grown in 12‐well plates to 95 ‐ 100% confluency. A/Shanghai/2/2013 H7N9 at a MOI of 0.1 was incubated with 200 nM matriptase/ST 14 in 200 µL matriptase buffer or with 0.8 nM trypsin in 200 µL flu infection medium (DMEM, 10 nM HEPES, 0.2% BSA) for 30 minutes at 37°C. For the negative control, virus was incubated in flu infection medium for 30 minutes at 37°C. Control, trypsin‐ and matriptase/ST 14‐treated virus were then added to one well MDCK cells each and incubated on a rocker for 45 minutes. After 45 minutes, 800 µL flu infection medium was added and cells were grown with virus for 48 hours. After 48 hours, supernatants were collected and stored at −80°C. Viral titers were analyzed using an immuno‐plaque assay as previously described.^18^ The experiment was repeated three independent times. Data and statistical analysis were performed using Graphpad Prism7 software.

## 3 RESULTS

### 3.1 H7N9 HA is cleaved by human matriptase/ST 14 and results in a fusogenic active protein

**Figure 1:**
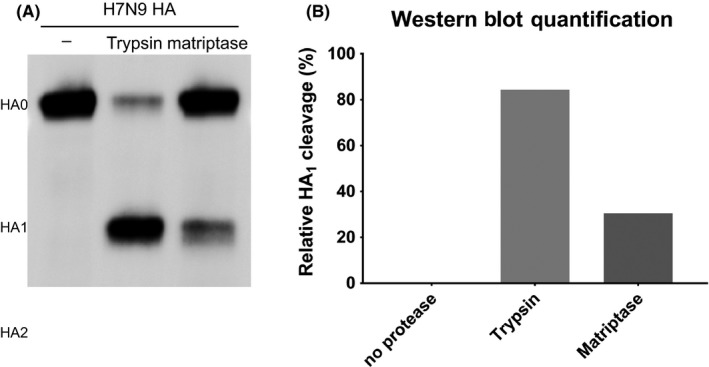
Analysis of matriptase/ST 14‐mediated H7N9 HA cleavage. H7N9 HA was expressed in 293T cells and incubated with no protease, trypsin, or matriptase/ST 14. (A) Western blot analysis of H7N9 HA cleavage. (B) Quantification of the percent HA cleavage from (A) using ImageJ software

We previously reported that recombinant human matriptase/ST 14 cleaved a fluorogenic peptide mimicking the cleavage site motif of A/Shanghai/2/2013 H7N9 HA very efficiently.^11^ Unlike the situation with other human‐specific proteases tested, the resulting Vmax value for matriptase/ST 14 was not significantly different from trypsin, which served as a positive control. However, results obtained in peptide assays using HA cleavage site mimics do not always translate into observations made with full‐length proteins.^11^, ^14^ Therefore, we expressed A/Shanghai/2/2013 H7N9 HA in 293T cells and subsequently added recombinant human matriptase/ST 14 for 90 minutes. No protease was added to the negative control and incubation with trypsin served as a positive control. We did not observe any cleavage of H7N9 HA without protease, and very efficient cleavage when trypsin was added (Figure 1A). Recombinant human matriptase/ST 14 cleaved H7N9, too, but to a lesser extent than trypsin (Figure 1A). Quantification revealed that trypsin converted about 80% of HA_0_ into HA_1_ and matriptase/ST 14 proteolytically processed about 30% HA_0_ into HA_1_ (Figure 1B).

**Figure 2:**
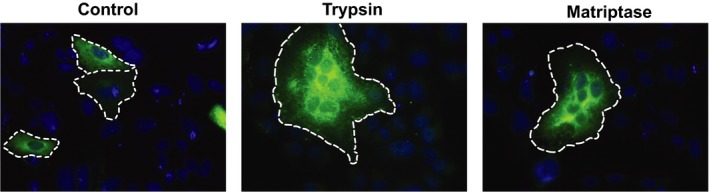
Analysis of H7N9 HA‐triggered cell‐cell fusion. Immunofluorescence staining of H7N9 HA without protease treatment and with trypsin and matriptase/ST 14 treatment. Single cells and syncytia‐forming cells are emphasized with a white dashed line. H7N9 HA was labeled with a specific 1st HA antibody and a secondary Alexa Fluor 488 antibody. Nuclei were stained with DAPI. Magnification of the images is 25×

Next, we were interested to see whether matriptase/ST 14‐mediated cleavage of H7N9 HA results in a fusogenic active protein. Therefore, we expressed H7N9 HA in VERO cells and incubated the cells subsequently with recombinant human matriptase/ST 14 for three hours. Controls were as described above. The negative control without added protease did not show cell fusion (Figure 2). However, when trypsin and matriptase/ST 14 were added to cells expressing H7N9 HA, strong syncytia formation was observed (Figure 2).

In conclusion, recombinant human matriptase/ST 14 proteolytically processes H7N9 HA that is then able to exert its fusogenic activity.

### 3.2 Viral entry is facilitated by matriptase/ST 14 cleaved H7N9 HA

After we established that recombinant human matriptase/ST 14‐mediated cleavage of results in a fusogenic H7N9 HA protein, we wanted to test whether it would promote the infectivity of the virus. As a first step, we chose to produce pseudoparticles representing surrogates of native virions that allowed us to study the viral entry into host cells. The pseudoparticles used in this assay possess a MLV core that incorporates a luciferase reporter gene upon their release from the producing cells.^17^ To facilitate fusion, the pseudovirions contained H7N9 HA and were incubated with trypsin or matriptase/ST 14 prior to their addition to cells. We used two negative controls: (a) pseudoparticles with H7N9 HA but no protease treatment and (b) pseudoparticles without a fusion protein. As additional control for the infectivity of the generated pseudoparticles, we used pseudovirions carrying the VSV‐G fusion protein that does not require proteolytical cleavage. Cells were lysed 48 hours after infection with the pseudoparticles, and the cell lysate was mixed with luciferase substrate. Measurement of the relative fluorescence provides a quantitative analysis of the infectivity.

**Figure 3:**
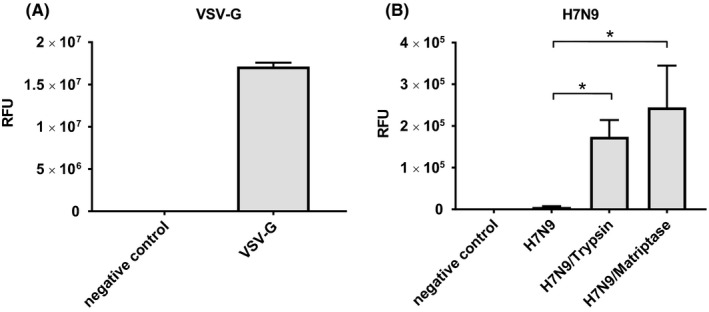
Infection with H7N9 pseudoparticles. Pseudoparticles carrying no fusion protein (negative control), VSV‐G, or H7N9 HA were produced in 293T cells and subsequently used to infect MDCK cells. H7N9 pseudoparticles were treated with no protease, trypsin, or matriptase/ST 14 prior to infection. (A) VSV‐G pseudoparticles served as control for the infectivity of the particles. (B) Infection studies with H7N9 HA carrying pseudoparticles. A two‐tailed *t* test was conducted to determine statistical significance of the untreated H7N9 control compared to trypsin and matriptase/ST 14‐treated H7N9 pseudoparticles. * = *P* < .02. RFU, relative fluorescence units

Pseudoparticles carrying VSV‐G were highly infective showing that the pseudoparticle production worked as expected (Figure 3A, Table 1A). H7N9 HA pseudovirions treated with trypsin and with matriptase/ST 14 were significantly more infectious compared to the same pseudoparticles without protease treatment (Figure 3B, Table 1A). However, there was no significant difference between trypsin‐ and matriptase/ST 14‐treated pseudoparticles suggesting that all pseudovirions were depleted (Figure 1B, Table 1A). Pseudoparticles without an incorporated fusion protein were not infectious. The data show that matriptase/ST 14‐mediated cleavage of H7N9 HA results in fully infectious virions that are able to fuse with host cells.

**Table 1:** Values from pseudoparticle infection (A) and A/Shanghai/2/2013 H7N9 infection (B) depicted in Figure 3 and Figure 4, respectively

(A)	Negative control	VSV‐G	H7N9	H7N9/Trypsin	H7N9/Matriptase

RFU	9.90E + 01	1.71E + 07	5.78E + 03	1.74E + 05	2.45E + 05
SD	1.87E + 01	4.54E + 05	1.74E + 03	4.03E + 04	1.00E + 05

(B)	No protease	Trypsin	Matriptase

pfu/mL	1.07E + 04	5.16E + 10	1.70E + 07
SD	3.79E + 03	1.54E + 10	1.00E + 06

### 3.3 Matriptase/ST 14 promotes viral growth of A/Shanghai/2/2013 H7N9

Next, we investigated whether matriptase/ST 14 was able to sustain and promote viral growth of A/Shanghai/2/2013 H7N9 in a cell culture system. Therefore, we infected MDCK cells with the live virus at a low MOI of 0.1 and added trypsin or matriptase/ST 14. The control did not receive protease treatment. After 48 hours, the supernatants were collected, and the viral titers were quantified using an immuno‐plaque assay.

**Figure 4:**
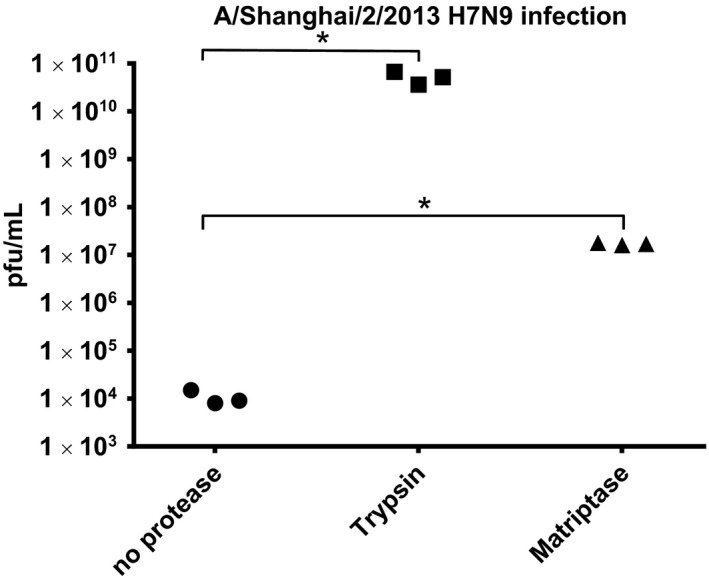
Infection with A/Shanghai/2/2013 H7N9 virus. MDCK cells were infected A/Shanghai/2/2013 H7N9 virus for 48 h, and viral titers in the supernatant were subsequently determined. Prior to infection, the virus was incubated with trypsin or matriptase/ST 14. Control did not receive protease treatment. Virus was then added to the cells including the respective protease. A two‐tailed *t* test was performed to determine *P*‐values of untreated control compared to trypsin and matriptase/ST 14‐treated samples. * = *P* < .01

Influenza A/Shanghai/2/2013 H7N9 that was not treated with a protease exhibited a titer of about 1 × 10^4^ pfu/mL (Figure 4, Table 1B). Virus that was treated with trypsin and matriptase/ST 14, however, had significantly higher titers of approximately 5 × 10^10^ pfu/mL and 1.7 × 10^7^, respectively (Figure 4, Table 1B).

Together, our data suggest that human matriptase/ST 14 can cleave H7N9 HA and may significantly contribute viral growth of influenza A/Shanghai/2/2013 H7N9 in humans.

## 4 DISCUSSION

Influenza H7N9 viruses have caused a significant number of causalities since their emergence in 2013 and pose a major threat for public health because of their ability to continuously evolve and reassort.^5^ This is well‐illustrated by the finding that H7N9 viruses from the 5th wave were antigenically distinct from the viruses that emerged in 2013, rendering existing candidate vaccines ineffective.^19^ Novel approaches to fight influenza infections include targeting host proteases that are responsible for the activation of the virus.^20^, ^21^, ^22^ A major benefit of this approach is that it is very unlikely to lead to resistance phenotypes in the virus. However, it requires the information by which proteases distinct influenza HA subtypes are proteolytically activated.

So far, the type II transmembrane serine protease TMPRSS2 is the only human protease that has been associated with the activation of LPAI H7N9 HA.^9^, ^10^ TMPRSS2 KO mice showed no clinical signs of disease and very limited spread of the virus when infected with A/Anhui/1/2013. However, the mice still exhibited low titers of virus several days post‐infection suggesting that other proteases are able to cleave LPAI H7N9 HA. Our data strongly suggest that matriptase/ST 14 has a major role in cleaving LPAI H7N9. The fact that TMPRSS2 KO mice did not show clinical signs of disease may not translate to human infections since there is no evidence that TMPRSS2 is the sole enzyme responsible for the spread of the virus in humans. Matriptase/ST 14 has been identified as one of the important host proteases cleaving HA directly in a subtype‐specific manner or indirectly by activating HA‐processing zymogens.^12^, ^13^, ^14^ To date, there are reports demonstrating matriptase/ST 14‐mediated cleavage of H1N1 and H9N2 HA. Matriptase/ST 14 also expresses selective HA cleavage for particular strains within the H1 subtype.^12^

In the context of our work, it is important to point out that a vast majority of human LPAI H7N9 strains share the same HA cleavage site motif as A/Shanghai/2/2013. We analyzed 1352 LPAI H7N9 sequences from human isolates collected between 2013 and 2019 that are available at the GISAID database (https://platform.gisaid.org/epi3/frontend#1001b7). Only seven sequences showed changes in the HA cleavage site motif; six strains exhibited a K to R substitution in the P3 position, and one strain had a K to Q change at the P3 position, too (data not shown). This emphasizes that requirements for virus activation largely remain the same even though antigenically different strains have evolved over the past 6 years. However, we have no data to predict if matriptase/ST 14 would be able to proteolytically process these changed cleavage sites.

We recently showed that matriptase/ST 14 cleaved a peptide representing the H7N9 LPAI HA cleavage motif very efficiently using an assay with fluorogenic peptides representing different HA subtype cleavage site mimics. A limitation of this assay is that peptides may produce false‐positive results because the structural interaction between protease and cleavage site might be altered compared to the full‐length HA protein. Matriptase/ST 14, for example, was reported to proteolytically process the A/Aichi/2/68 H3N2 HA cleavage site motif in peptide assays.^12^, ^14^ When matriptase/ST 14 was then tested with the A/Aichi/2/68 H3N2 HA full‐length protein expressed in cells, only little cleavage that did not produce fusogenic HA or no cleavage was observed. Here we demonstrate that human matriptase/ST 14 cleaves H7N9 HA expressed in cells resulting in a fully fusogenic active protein validating our results from the previously published peptide assay. Matriptase/ST 14‐mediated cleavage of H7N9 HA also promoted the infectivity of pseudovirions and A/Shanghai/2/2013 virus in cell culture. Our data indicate that human matriptase/ST 14 is a relevant protease to cause LPAI H7N9 infectivity in humans and may represent an important pathogenicity determinant and target for therapeutic intervention.
